# Merkel Cell Polyomavirus DNA Detection in Respiratory Samples: Study of a Cohort of Patients Affected by Cystic Fibrosis

**DOI:** 10.3390/v11060571

**Published:** 2019-06-21

**Authors:** Carla Prezioso, Federica Maria Di Lella, Donatella Maria Rodio, Camilla Bitossi, Maria Trancassini, Annamaria Mele, Corrado de Vito, Guido Antonelli, Valeria Pietropaolo

**Affiliations:** 1 Department of Public Health and Infectious Diseases, Sapienza University, 00185 Rome, Italy; carla.prezioso@uniroma1.it (C.P.); dona.rodio@gmail.com (D.M.R.); maria.trancassini@uniroma1.it (M.T.); annamaria.mele@uniroma1.it (A.M.); corrado.devito@uniroma1.it (C.d.V.); 2 Department of Experimental Medicine, University of Campania Luigi Vanvitelli, 80138 Naples, Italy; federicamariadilella@gmail.com; 3 Department of Molecular Medicine and Pasteur Institute-Cenci Bolognetti Foundation, Sapienza University, 00161 Rome, Italy; camillabitossi1@gmail.com (C.B.); guido.antonelli@uniroma1.it (G.A.)

**Keywords:** Merkel cell polyomavirus, cystic fibrosis, VP1, sequence alignment, phylogenetic analysis, *Staphylococcus aureus*

## Abstract

Background: The role of Merkel cell polyomavirus (MCPyV) as a respiratory pathogen is controversial, and it is still unclear in patients with cystic fibrosis (CF). The aim of this study was to define the MCPyV prevalence and epidemiology in CF patients in order to gain new insights into the association between MCPyV infection and respiratory diseases. Methods: A one-year study was conducted testing oropharyngeal aspirate samples from 249 and 124 CF and non-CF patients, respectively. Detection of MCPyV was carried out by nested polymerase chain reaction (PCR). Moreover, a sequence alignment to examine viral capsid protein 1 (VP1) and a phylogenetic analysis were performed. Results: MCPyV DNA was detected in 65 out of 249 samples analyzed CF (26%), a percentage that was higher than that recorded in non-CF patients (0.8%). There were no statistically significant differences in MCPyV prevalence according to gender, while there was a correlation between MCPyV detection and age. Interestingly, an association between the presence of MCPyV and the concurrent isolation of *Staphylococcus aureus* was found. Sequence analysis of MCPyV VP1 and phylogenetic analysis revealed a 99% homology with the published sequences of these viruses in GenBank. Conclusions: Detection of MCPyV in CF patient specimens pointed out a possible interaction between the virus and CF. Further studies are necessary to fully understand the involvement of MCPyV in the pathogenesis of respiratory disorders.

## 1. Introduction

Merkel cell polyomavirus (MCPyV) is a small, nonenveloped, double-stranded DNA virus identified in 2008 [1]. Most MCPyV infections are asymptomatic, and serological studies showed that 50–80% of the healthy adult population present MCPyV-specific antibody responses [2,3]. Since MCPyV seroconversion becomes prevalent in the first decade of a child’s life and continues to increase with age, it has been hypothesized that primary infection occurs early in childhood. The route of transmission and the sites of the initial and latent infections have not been understood. Feng and co-workers found MCPyV DNA integrated clonally in the genome of Merkel cell carcinomas, supporting the hypothesis that viral infection may be an early event in the pathogenesis of Merkel cell carcinoma (MCC) [1]. MCPyV was found in healthy skin and rarely concludes with MCC. These observations support the hypothesis that MCPyV itself on the skin is not a sufficient requirement for MCC carcinogenesis [4].

Moreover, MCPyV DNA has been detected in a variety of non-MCC cancers, including chronic lymphocytic leukemia, malignant tonsillar tissues, cervical carcinomas, nonmelanoma skin cancers, and lung cancer [5,6,7,8,9,10,11]. More recent studies have detected MCPyV in various specimens, including respiratory tract samples [12,13,14,15]. It is well documented that MCPyV can infect the upper and lower respiratory tract [12,13,14,15,16], and MCPyV found in respiratory secretions is analogous to the virus identified in MCC [17]. It has been assumed that MCPyV is an etiological agent behind MCC and, possibly, of other human cancers [18], but its role in respiratory disease has not been established. In this framework, the pathogenic role of polyomaviruses (PyVs), such as BKPyV and JCPyV, is well described in immunosuppressed people [19,20,21], while the specific role of MCPyV is still debated. To date, only limited studies evaluated the prevalence of MCPyV in patients suffering from cystic fibrosis (CF), a well-known multiorgan genetic disorder characterized by recurrent pulmonary exacerbation [9] mainly caused by chronic bacterial infections, although the role of viral infections has not been ruled out [22,23,24]. To gain new insights into a potential pathogenic role played by MCPyV in CF, we investigated the presence of MCPyV DNA in the upper respiratory tract of CF patients. Last, since our understanding of MCPyV phylogenetic characteristics is still poor, a further objective was to examine the properties of MCPyV strains circulating in a CF cohort.

## 2. Materials and Methods

### 2.1. Patients and Sample Collection

A cross-sectional study including 249 CF patients—97 were males (39%) and 152 were females (61%) with ages between 1 and 62 years (mean age: 18.4 years; median age: 14 years)—already diagnosed and enrolled at the CF Reference Center of the Lazio Region Policlinico Umberto I Hospital (Rome, Italy) was performed. From this cohort, a total of 249 oropharyngeal aspirate samples was collected from January to December 2018.

The CF cohort of patients was divided arbitrarily into three subcohorts with respect to age: children (≤6 years), older children (between 7 and 18 years), and adults (>18 years). In detail, 48 children (19.3%) (mean age: 3.9 years), 113 older children (45.4%) (mean age: 12 years), and 88 adults (35.3%) (mean age: 34.4 years) were enrolled. Relatively to the gender, children were represented by 32 females and 16 males, older children by 65 females and 48 males, whereas adults included 55 females and 33 males.

Moreover, the study was also performed on 124 individuals not affected by CF (non-CF)—51 males (41.1%) and 73 females (58.9%) with ages between 1 and 63 years (mean age: 18.7 years, median age: 14.5 years)—who were admitted to the Policlinico Umberto I Hospital with a suspected diagnosis of acute respiratory disease. From this non-CF cohort, in the same period, 124 oropharyngeal aspirate samples were collected.

The division into three subcohorts with respect to age was maintained: 24 children (19.3%) (mean age: 4.4 years), 56 older children (45.1%) (mean age: 12.7 years), and 44 adults (35.4%) (mean age: 34.1 years). In relation to the gender, children were represented by 15 females and 9 males, older children by 32 females and 24 males, whereas adults included 26 females and 18 males.

The study was approved by the local Ethic Committee (Ethic Committee Sapienza University of Rome, Policlinico Umberto I Hospital) N 5223 (approval date 25 October 2018), and informed consent was obtained from patients or their parents when necessary. All samples were tested for routine microbiological analysis with cultures for common and emerging microorganisms by using appropriate media.

### 2.2. Merkel Cell Polyomavirus (MCPyV) Polymerase Chain Reaction (PCR)

Strict procedures were utilized to prevent cross-contamination during the experiments including the use of separate plasmid-free rooms and centrifuges for clinical sample processing.

DNA was extracted using DNeasy^®^ Blood and Tissue Kit (QIAGEN, S.p.A, Italy) according to the manufacturer’s instructions. To test DNA quality, β-globin PCR was carried out as previously described [25]. All β-globin-positive specimens were tested for MCPyV DNA by a nested PCR using primers targeting MCPyV viral capsid protein 1 (VP1), as previously described [26].

The plasmid pMCV-R17a containing the complete genome of MCPyV (Addgene, Cambridge, MA, USA) was used as a positive control, and distilled water was used as a negative control.

By electrophoresis in 2% agarose gel, PCR products were analyzed stained with ethidium bromide and observed under UV light. Using tenfold serial dilutions of positive controls, VP1 PCR sensitivity was determined as 50 copies/mL. All MCPyV-positive specimens were retested. Standard precautions were adopted in order to prevent contamination during PCR setup and PCR analysis. A negative control was included in each set of PCR.

### 2.3. MCPyV DNA Sequencing

PCR products corresponding to the VP1 region were purified with a QIAquick^®^ PCR purification kit, according to QIAGEN protocol. DNA sequencing was performed using a dedicated facility (Bio-Fab research s.r.l., Rome, Italy).

### 2.4. Viral Capsid Protein 1 (VP1) Alignment and Phylogenetic Analysis

In order to study the MCPyV VP1 sequences, 10 CF representative oropharyngeal aspirate samples were compared with reference sequences present in GenBank (EU375803 and EU375804). Sequence alignment was obtained using ClustalW2 on the European Molecular Biology Laboratory–European Bioinformatics Institute (EMBL-EBI) website using default parameters [27]. Phylogenetic analysis was carried out on VP1 sequences derived from the same 10 oropharyngeal samples, MCPyV reference sequences EU375803 and EU375804, and representative BKPyV, JCPyV, KIPyV, and WUPyV sequences obtained from GenBank. The phylogenetic tree was generated using Molecular Evolutionary Genetics Analysis (Mega) version 6.0 software using the neighbor joining algorithm [28]. A bootstrap test with 1000 replicates was performed to evaluate the confidence of the branching pattern of the trees.

### 2.5. Statistical Analysis

MCPyV detection was summarized by counts and proportions. If continuous variables were normally distributed, they were expressed as mean ± SD; if not, they were expressed by median and range. The *χ*^2^ test was performed to evaluate differences in the viral detection in the cohorts of study, and the Mann–Whitney U-test for non-normally distributed continuous variables was applied to analyze differences between patients. A *p* value less than 0.05 was considered statistically significant.

## 3. Results

Two hundred forty-nine and 124 oropharyngeal aspirate samples collected from CF and non-CF patients, respectively, were randomly collected from January to December 2018 and analyzed for MCPyV DNA presence. No statistically significant differences in relation to the age and gender were observed (not significant, NS).

MCPyV DNA was detected in 65/249 CF specimens (26%) comprising 43 females (66%) and 22 males (34%) (Table 1). In particular, in the subcohort of children, MCPyV DNA was detected in 11/48 (23%) samples comprising 7 females and 4 males. In the subcohort of older children, MCPyV DNA was present in 28/113 (25%) samples comprising 17 females and 11 males. Among adults, 26/88 subjects (30%) comprising 19 females and 7 males were positive for MCPyV DNA. No MCPyV infection related to gender was observed (NS) (Table 1).

Regarding a possible linkage between MCPyV DNA-positive samples and CF patient age, no significant associations were observed in the subcohort of children (NS) and older children (NS) (Table 1). Differently, a statistically significant difference was found in the subcohort of adults (*p* = 0.037) (Table 1).

In the non-CF cohort of respiratory patients, results revealed that 11/124 samples (9%) were positive for MCPyV DNA. In detail, 6 belonged to the female sex (1 child, 2 older children, and 3 adults), while 5 belonged to the male sex (1 child, 2 older children, and 2 adults) (Table 1). No statistically significant difference was found in MCPyV prevalence according to age or gender (NS) (Table 1).

It is well-known that CF patients are frequently colonized by different bacterial pathogens. Within the respiratory samples of the CF cohort, the most common pathogens detected were *Staphylococcus aureus* and *Pseudomonas aeruginosa.* Analysis of the simultaneous presence of MCPyV in a CF cohort of patients with respect to *S. aureus* and *P. aeruginosa* revealed that 47/65 (72%) were colonized by *S. aureus*, with a statistically significant correlation (*p* = 0.001), whereas 12/65 (19%) were colonized by *P. aeruginosa* without a significant association (NS) (Table 2). Specifically, the co-presence of MCPyV and *S. aureus* was significant in the subcohorts of children (*p* = 0.030) and older children (*p* = 0.001) but not in the subcohort of adults (NS) (Table 2). Analysis of prevalence of MCPyV infection with respect to *P. aeruginosa* colonization showed no statistically significant correlation within the three subcohorts (NS) (Table 2). To corroborate the hypothesis that the environment present in the lungs of CF patients is favorable to MCPyV infection/reactivation and to bacteria colonization, and to provide global information about the differences in MCPyV positivity rate between CF and non-CF patients, a single database of 373 samples collected from 249 CF patients and 124 non-CF patients was created. No statistically significant difference was observed between CF and non-CF patients in relation to age and gender (NS). Nevertheless, it is noteworthy to highlight that a statistically significant association was found between MCPyV presence and CF disease (*p* = 0.001) and between CF disease and *S. aureus* (*p* = 0.001) (Table 3).

**Figure 1:**
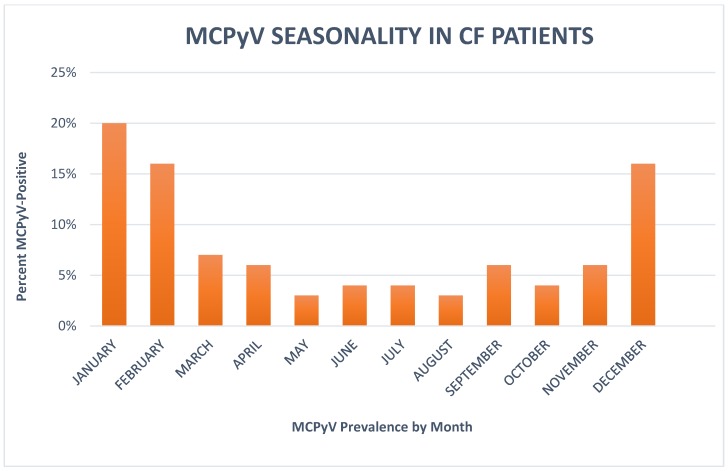
Bars represent the percentages (%) of Merkel cell polyomavirus (MCPyV)-positive samples. The percent of MCPyV-positive cases was observed predominantly in December, January, and February, with highest detection in January (20% of the month’s samples). CF = cystic fibrosis.

In order to verify the trend of MCPyV infection in CF patients during the year, data were examined taking into account the month of virus detection. MCPyV was observed every month, with the greatest number of positive cases in January, February, and December. The highest prevalence was observed in January (20% of the month’s samples), suggesting a winter seasonality (Figure 1).

**Figure 2:**
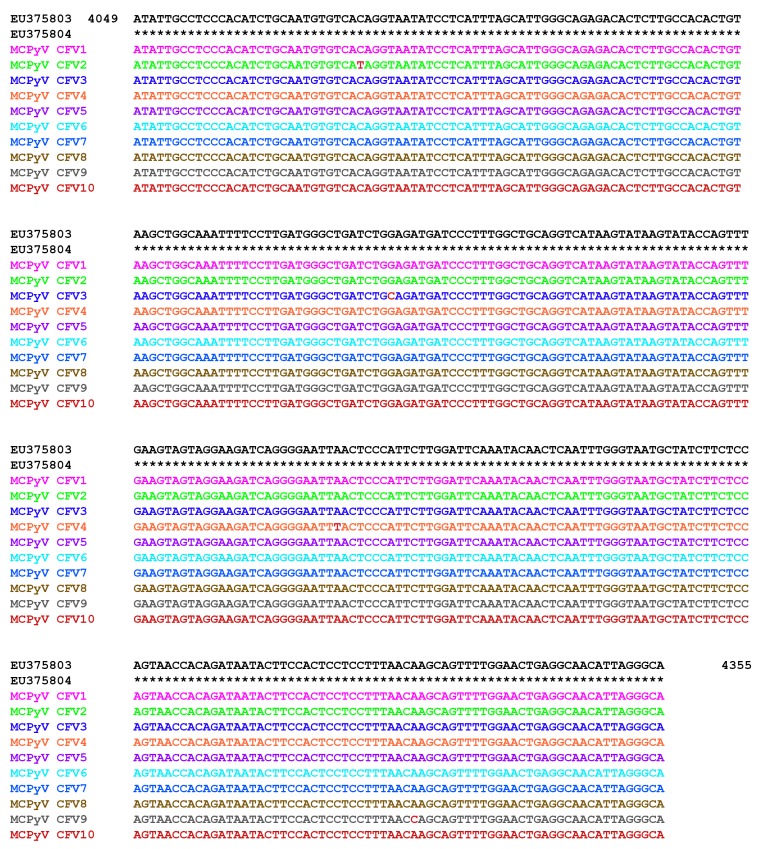
Sequence analysis of the MCPyV viral capsid protein 1 (VP1) PCR products. An alignment is shown between the nucleotide sequence from 4049 to 4355 of the published sequences of MCPyV in GenBank (NCBI) (EU375803; EU375804) [1] and that obtained from the sequencing of oropharyngeal aspirate samples positive for MCPyV VP1 (CFV1 to CFV10). The sequence product study revealed 99% homology with the published sequences. Nucleotide substitutions are highlighted in bold.

**Figure 3:**
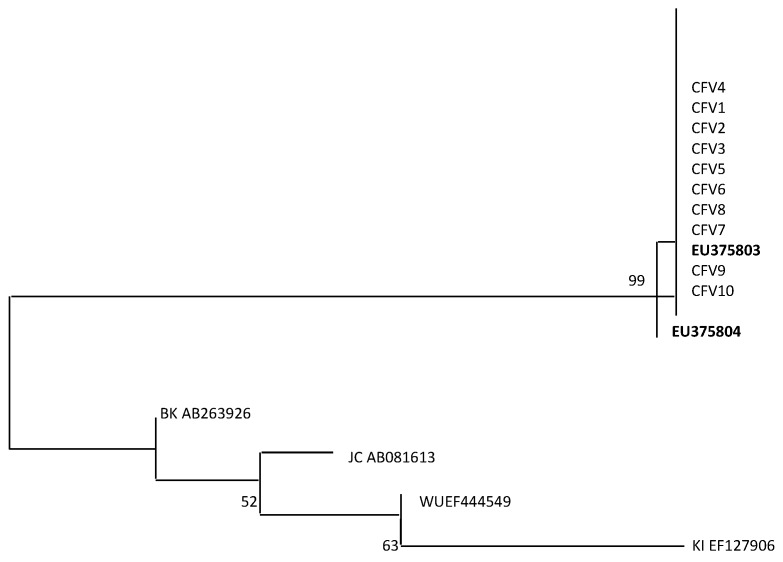
Phylogenetic analysis of nucleotide sequences of MCPyV VP1 from 10 CF representative oropharyngeal aspirate samples. Phylogenetic tree including the VP1 sequences CFV (1 to 10). The phylogenetic tree has been generated by retrieving two MCPyV reference strain sequences deposited in Genbank (NCBI) under the following accession numbers: EU375803 and EU375804. Moreover, the analysis includes representative BKPyV, JCPyV, KIPyV, and WUPyV sequences from GenBank. The phylogenetic tree was generated using Mega 6.0 software using the neighbor joining algorithm. Bar, 0.02 substitutions per site.

Finally, in order to study the properties of MCPyV strains circulating in a CF cohort, a MCPyV VP1 alignment was carried out comparing 10 CF representative oropharyngeal aspirate samples and reference strains EU375803 and EU375804 [1]. Results revealed a 99% homology with the deposited sequences of MCPyV in GenBank (Figure 2). Furthermore, a phylogenetic analysis including MCPyV reference sequences EU375803 and EU375804, the same 10 oropharyngeal aspirate samples, and representative BKPyV, JCPyV, KIPyV, and WUPyV sequences (NCBI) showed that all CF isolated strains fell into the same cluster (Figure 3).

## 4. Discussion

Progressive pulmonary disease is the primary cause of morbidity and mortality in CF patients. In recurrent pulmonary exacerbation, the role of chronic bacterial infections is well-known, whereas the role of viral infections is still debated. Although the presence of WUPyV and KIPyV in respiratory samples of hematology/oncology [29] and AIDS [30] patients has been demonstrated, to our knowledge, only one study evaluated the prevalence of HPyV in CF patients [16].

On these bases, our main aim was to assess the prevalence of MCPyV in CF respiratory secretions to further address the potential pathogenic role of MCPyV in CF and to understand if this genetic disease may be associated to MCPyV infection and/or reactivation.

Iaria et al. [16] showed that CF patients with underlying respiratory diseases could develop a greater susceptibility to PyV infection. Our results consolidated the hypothesis that the respiratory tract may be considered a route of transmission for MCPyV, which has already been demonstrated for JCPyV and BKPyV [21]. Moreover, our data showed a remarkable prevalence of MCPyV DNA higher than those present in literature [13,15,16].

Two main considerations could be taken into account to support these findings: first, the high rate of MCPyV detection could be due to the microenvironment present in CF patient lungs, supporting MCPyV infection and or reactivation; second, in our experimental workflow, an efficient nested PCR [26] was performed. This methodological approach, targeting the MCPyV VP1 region, is characterized by a higher sensitivity and a higher frequency of virus detection (<200 copies/mL) compared to PCR that targets the large T-antigen gene and whose sensitivity is 1000 copies/mL [15].

The evidence of age-related MCPyV prevalence could be explained assuming that, in the case of children younger than 6 years, the respiratory system could be a primary target for MCPyV. Subsequently, MCPyV can spread through the respiratory route to other body compartments, establishing a state of latency. In the case of patients >18 years, the significant higher prevalence of MCPyV could be clarified, supposing an association between MCPyV and susceptible patients, as patients with chronic disease, including CF patients.

Regarding the bacterial colonization status in CF, it is well-known that, among the most common respiratory pathogens, *S. aureus* and *P. aeruginosa* show a range of competitive and cooperative interactions. Our data highlight that MCPyV detection was significantly associated with the presence of *S. aureus* within 47/65 MCPyV-positive CF samples and, in particular, in the subcohorts of children and older children. A careful analysis of the literature supports our results emphasizing that *S. aureus* is the most frequent respiratory pathogen in infants and young children with CF [31,32]. In contrast, the absence of a significant association between MCPyV and *P. aeruginosa* could be due to the lower incidence of this infection in CF patients [31,32].

The inverse patterns of colonization represented by *S. aureus* and *P. aeruginosa* allowed us to hypothesize that competition between these organisms prevents their copresence during infection within CF disease. It is tempting to speculate that *S. aureus* could contribute to pulmonary inflammatory processes, inducing MCPyV infection and or reactivation. MCPyV replication and concomitant immunosuppression, representative of CF patients, may cause pulmonary damage and facilitate *S. aureus* colonization. Consequently, our data further corroborate the hypothesis that a specific environment, particular to the lungs of CF patients, is favorable for MCPyV infection/reactivation and facilitates bacteria colonization.

In regard to the viral seasonal trend, a higher frequency of MCPyV was observed predominantly in December, January, and February, with highest detection in January, suggesting that the virus could be transmitted more frequently during cold months, like other respiratory viruses, or that cold temperatures could favor viral replication in respiratory tissues.

Finally, our data regarding VP1 alignment and phylogenetic analysis showed that all CF sequences isolated were similar (99%) to the virus sequence identified within MCC [1] and fell into the same cluster. Although nucleotide substitutions or deletions in structural genes of other HPyVs have been described, in our study we detected minor changes in the nucleotide order of the VP1 sequence (<1%). This result suggests a stability and a similarity across different isolates of the MCPyV VP1. Our study adds, in our opinion, important knowledge on the issue of MCPyV prevalence in CF patients.

The high level of MCPyV DNA detection suggests that a relationship between this virus and CF disease may exist, and CF could favor replication of MCPyV and other latent or ubiquitous viruses by unknown mechanisms. Further studies are necessary to fully understand the involvement of MCPyV in respiratory disorders and the role of viral infections in CF lung disease.

## Figures and Tables

**Table 1:** MCPyV DNA detection in the enrolled CF patients and in non-CF patients.

Subject Category	CF Patients *N* = 249	*p*-Value	Non-CF Patients *N* = 124	*p*-Value
Age, mean ± SD	1–62, 18.4 ± 14.3	*NS*	1–63,18.7 ± 14	*NS*
Age ≤ 6 years, n (%)	48 (19%)		24 (19%)	
Age 6–17 years, n (%)	113 (45%)		56 (45%)	
Age > 18 years, n (%)	88 (35%)		44 (35%)	
Sex, n female (%)	152 (61%)	*NS*	73 (59%)	*NS*
Among ≤ 6 year olds, n (%)	32 (21%)		15 (8%)	
Among 7–17 year olds, n (%)	65 (43%)		32 (7%)	
Among >18 year olds, n (%)	55 (36%)		26 (11%)	
Sex, n male (%)	97 (39%)	*NS*	51 (41%)	*NS*
Among ≤ 6 year olds, n (%)	16 (17%)		9 (18%)	
Among 7–17 year olds, n (%)	48 (49.4%)		24 (47%)	
Among >18 year olds, n (%)	33 (34%)		18 (35%)	
MCPyV-positive, n (%)	65 (26%)		11 (9%)	
Among ≤ 6 year olds, n (%)	11 (23%)	*NS*	2 (8%)	*NS*
Among 7–17 year olds, n (%)	28 (25%)	*NS*	4 (7%)	*NS*
Among >18 year olds, n (%)	26 (30%)	*p* = 0.037	5 (11%)	*NS*
MCPyV-positive, n (%) female	43 (66%)	*NS*	6 (8%)	*NS*
Among ≤ 6 year olds, n (%)	7 (22%)		1 (7%)	
Among 7–17 year olds, n (%)	17 (26%)		2 (6%)	
Among >18 year olds, n (%)	19 (34%)		3 (11%)	
MCPyV-positive, n (%) male	22 (34%)	*NS*	5 (10%)	*NS*
Among ≤ 6 year olds, n (%)	4 (25%)		1 (1%)	
Among 7–17 year olds, n (%)	11 (23%)		2 (8.3%)	
Among >18 year olds, n (%)	7 (21%)		2 (4%)	

CF: cystic fibrosis; MCPyV: Merkel cell Polyomavirus; n: number; NS: not significant.

**Table 2:** MCPyV, *Staphylococcus aureus*, and *Pseudomonas aeruginosa* colonization status in CF patients.

Subject Category	CF Patients *N* = 249	*S. aureus* Colonization Status	*p*-Value	*P. aeruginosa* Colonization Status	*p*-Value
MCPyV-positive, n (%)	65 (26%)	47 (72%)	*p* = 0.001	12 (18.5%)	*NS*
Among ≤ 6 year olds, n (%)	11 (23%)	9 (19%)	*p* = 0.030	2 (16%)	*NS*
Among 7–17 year olds, n (%)	28 (25%)	18 (38%)	*p* = 0.001	3 (25%)	*NS*
Among >18 year olds, n (%)	26 (30%)	20 (43%)	*NS*	7 (59%)	*NS*

CF: cystic fibrosis; MCPyV: Merkel cell Polyomavirus; *S. aureus: Staphylococcus aureus; P. aeruginosa: Pseudomonas aeruginosa;* n: number; NS: not significant.

**Table 3:** Association of CF disease with MCPyV DNA presence and *Staphylococcus aureus* colonization status.

**Subject Category**	**CF Patients *N*= 249**	***p*-Value**	***S. aureus* Colonization Status**	***p*-Value**
MCPyV-positive, n (%)	65 (26%)	*p* = 0.001	47	*p* = 0.001
	**non-CF patients N= 124**	***p*-value**	***S. aureus* colonization status**	***p*-value**
MCPyV-positive, n (%)	11 (9%)	*NS*	12	*NS*

CF: cystic fibrosis; MCPyV: Merkel cell Polyomavirus; *S. aureus: Staphylococcus aureus;* n: number. NS: not significant.
